# Accuracy of radiomics in the diagnosis and preoperative high-risk assessment of endometrial cancer: a systematic review and meta-analysis

**DOI:** 10.3389/fonc.2024.1334546

**Published:** 2024-01-25

**Authors:** Junmei He, Yurong Liu, Jinzhu Li, Shuang Liu

**Affiliations:** The Fifth People’s Hospital of Jinan, Jinan, Shandong, China

**Keywords:** radiomics, endometrial cancer, endometrial malignancy, lymph node metastasis, systematic review

## Abstract

**Background:**

With the increasing use of radiomics in cancer diagnosis and treatment, it has been applied by some researchers to the preoperative risk assessment of endometrial cancer (EC) patients. However, comprehensive and systematic evidence is needed to assess its clinical value. Therefore, this study aims to investigate the application value of radiomics in the diagnosis and treatment of EC.

**Methods:**

Pubmed, Cochrane, Embase, and Web of Science databases were retrieved up to March 2023. Preoperative risk assessment of EC included high-grade EC, lymph node metastasis, deep myometrial invasion status, and lymphovascular space invasion status. The quality of the included studies was appraised utilizing the RQS scale.

**Results:**

A total of 33 primary studies were included in our systematic review, with an average RQS score of 7 (range: 5–12). ML models based on radiomics for the diagnosis of malignant lesions predominantly employed logistic regression. In the validation set, the pooled c-index of the ML models based on radiomics and clinical features for the preoperative diagnosis of endometrial malignancy, high-grade tumors, lymph node metastasis, lymphovascular space invasion, and deep myometrial invasion was 0.900 (95%CI: 0.871–0.929), 0.901 (95%CI: 0.877–0.926), 0.906 (95%CI: 0.882–0.929), 0.795 (95%CI: 0.693–0.897), and 0.819 (95%CI: 0.705–0.933), respectively.

**Conclusions:**

Radiomics shows excellent accuracy in detecting endometrial malignancies and in identifying preoperative risk. However, the methodological diversity of radiomics results in significant heterogeneity among studies. Therefore, future research should establish guidelines for radiomics studies based on different imaging sources.

**Systematic review registration:**

https://www.crd.york.ac.uk/PROSPERO/display_record.php?RecordID=364320 identifier CRD42022364320.

## 1 Introduction

Endometrial cancer (EC), also known as uterine corpus cancer, is the most prevalent gynecological cancer in high-income countries, with an increasing global incidence rate. This cancer mainly occurs in perimenopausal and postmenopausal women. Current lifestyle leads to the rising prevalence of obesity, thereby increasing the risk of EC (1–3). The annual mortality risk for EC patients exhibits an upward trend (4).

Surgical intervention remains the primary treatment approach for EC patients, with total hysterectomy combined with bilateral salpingo-oophorectomy being the standard procedure. Surgery can be performed through open or minimally invasive methods. Some EC patients may require adjuvant therapies (4). There is considerable variation in the postoperative prognosis of EC patients. Two reviews have indicated that tumor diameter, myometrial invasion, lymphovascular space invasion, and lymph node metastasis are significantly associated with poor prognosis (5, 6). However, in clinical practice, achieving an accurate preoperative diagnosis of high-grade tumors, myometrial invasion, lymph node metastasis, and lymphovascular space invasion remains a daunting challenge.

Radiomics, an emerging field based on quantitative imaging techniques, can extract high-throughput quantitative radiological features from medical images (7, 8). Radiomics is the process of extracting quantifiable features from large amounts of data that may be relevant to potential biological or clinical outcomes using advanced machine learning analysis techniques. It is carried out based on 2D, 3D or 4D medical images. There are two main branches of the field, namely, manual radiomics and deep learning radiomics (9). In manual radiomics, firstly, clinical staff use specialized software (commonly 3D-slicer (10) or ITK-snap (11)) to segment the region of interest (ROI) and extract texture features in the ROI region from established medical images. Secondly, in the process of feature filtering or dimensionality reduction, a large number of features will be generated in the process of extracting texture features of the ROI region, thus creating a “dimensionality disaster”. Therefore, it is necessary to combine with reasonable feature filtering methods or dimensionality reduction methods (e.g., principal component analysis). Thirdly, machine learning models (e.g., Random Forest, Support Vector Machines, Artificial Neural Networks) are then constructed based on the filtered features or dimensionality reduction results to make predictions about disease state or treatment outcomes. Fourthly, the constructed machine learning method is then validated. As for radiomics carried out by deep learning, researchers can directly construct deep learning models based on images (12, 13). Currently, manual radiomics is dominant in radiomics research.

Radiomics has gradually been used to help develop tumor treatment strategies (14). Against this backdrop, radiomics has been introduced into the detection of endometrial malignancies and the preoperative risk assessment of EC patients. However, comprehensive systematic evidence is required to explore its feasibility and accuracy. Therefore, the present study was carried out to investigate the application value of radiomics in the diagnosis and treatment of EC patients.

## 2 Methods

### 2.1 Study registration

The current study was conducted following the Preferred Reporting Items for Systematic Reviews and Meta-analyses (PRISMA) statement (15). The study protocol has been registered in the International prospective register of systematic reviews (ID: CRD42022364320).

### 2.2 Eligibility criteria

#### 2.2.1 Inclusion criteria

1. Studies that had reasonable diagnostic criteria for (EC).
2. Studies that comprehensively constructed radiomics-based machine learning (ML) models for the detection of endometrial malignancy or risk assessment, including high-grade EC, lymph node metastasis, deep myometrial invasion, and lymphovascular space invasion.
3. Primary studies in which independent external validation was not performed.
4. Studies that used different ML approaches published on the same dataset.
5. Study designs: case-control studies, cohort studies, cross-sectional studies, or randomized controlled trials (RCTs).
6. Studies reported in English.

#### 2.2.2 Exclusion criteria

1. Meta-analyses, reviews, expert opinions, guidelines, and similar types of studies.
2. Studies that only conduct differential factor analysis without constructing complete ML models.
3. Studies lacking the following outcome measures for evaluating the predictive accuracy of ML models: ROC curve, c-statistic, c-index, sensitivity, specificity, accuracy, recall, precision, confusion matrix, diagnostic fourfold table, F1 score, and calibration curve.
4. Studies with a small sample size (<20 cases).
5. Studies that solely focused on image segmentation or texture extraction without constructing complete ML models.

### 2.3 Data sources and search strategy

PubMed, Cochrane, Embase, and Web of Science databases were retrieved up to July 24, 2022. A combination of MeSH terms and free-text terms was used for the search, without restrictions on publication year or region. To mitigate the risk of missing newly published primary studies, we conducted additional searches in all databases in March 2023. Detailed search strategies are presented in **Supplementary Table 1**.

### 2.4 Study selection and data extraction

The retrieved articles were imported into EndNote software. Duplicate studies were identified and excluded using both automated and manual methods. Titles and abstracts were screened to select potentially eligible studies. Full texts of these articles were then downloaded and read to determine eligible primary studies.

Prior to data extraction, a standardized form was used to collect the following information: first author, country, year of publication, type of artificial intelligence model, sample size, mean/median age of patients, histological grading of EC, depth of myometrial invasion and cervical invasion, assessment of lymph node metastasis, source of imaging data, number of segmenters for ROI segmentation and software used, number of cases in the training set, generation method of validation set, number of cases in the validation set, feature selection method, model type, modeling variables, and outcome measures for model evaluation.

The aforementioned literature screening was conducted independently by two researchers, with cross-checking performed upon completion. Discrepancies, if any, were resolved by consulting a third researcher.

### 2.5 Assessment of study quality

The methodological quality of the included studies was appraised by two independent researchers using the Radiomics Quality Score (RQS) (8). After completion, a cross-check was carried out. Dissents, if any, were resolved by consulting a third researcher.

### 2.6 Outcomes

The primary outcome measure is the c-index, which reflects the overall accuracy of ML models. In many primary studies, only the c-index was reported. However, when the number of cases is severely imbalanced, it becomes challenging to interpret the specific accuracy of the model for positive and negative events based on the c-index alone. Therefore, our main outcome measures also include sensitivity and specificity at the optimal threshold value of the model.

### 2.7 Synthesis methods

A meta-analysis of c-index was carried out to assess the overall accuracy of the ML models. For primary studies where the 95% confidence interval and standard error were missing for the c-index, we estimated the standard error following the approach described by Debray et al. (16). A random-effects model was preferred for the meta-analysis of c-index, given the variations in the included variables and inconsistent parameters across different ML models.

Additionally, the meta-analysis of sensitivity and specificity was performed utilizing a bivariate mixed-effects model. The meta-analysis of sensitivity and specificity was based on the diagnostic fourfold table. However, as many primary studies did not report the diagnostic fourfold table, we constructed it using sensitivity, specificity, precision, and the number of cases, or using sensitivity and specificity derived from the best Youden’s index and the number of cases. R4.2.0 was employed for meta-analysis (R development Core Team, Vienna, http://www.R-project.org).

## 3 Results

### 3.1 Study selection

**Figure 1:**
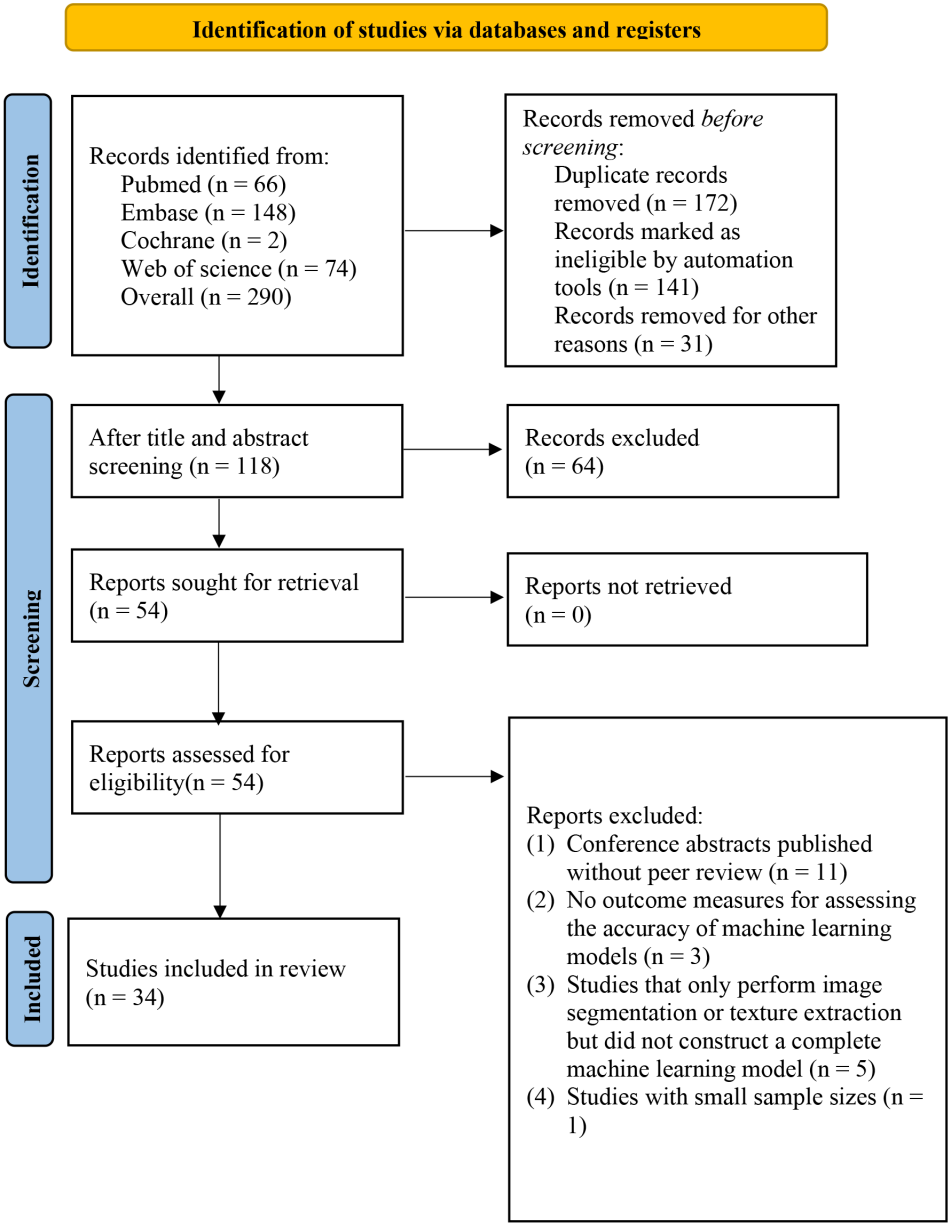
Flowchart of literature screening.

We retrieved a total of 290 articles (183 from the initial search and 107 from the supplementary search), out of which 172 were identified as duplicates (141 by automated software and 31 by manual identification). After screening titles and abstracts, 54 articles remained. After reading the full text, 33 studies were ultimately included in our systematic review (17–49) (**Figure 1**).

### 3.2 Study characteristics

This study includes 33 articles published within the past five years. These studies were conducted in countries such as Italy, France, Norway, China, Spain, Japan, and Canada. Among the 33 studies included, two studies (18, 32) were prospective cohort studies, while the other studies were case-control studies. Eleven studies (24, 30, 35, 36, 38, 41, 42, 44–46, 48) were multicenter studies, while the other 23 studies were conducted at a single center. Three studies (17, 21) utilized 18F-FDG PET/CT as the imaging modality, while the rest of the studies utilized MRI. The predominant models in the included studies were logistic regression (LR), with only a few studies utilizing artificial neural networks (ANN), support vector machines (SVM), and decision trees (DT). Detailed information on the included studies is provided in **Supplementary Table 2**.

### 3.3 Assessment of study quality

The included primary studies achieved no scores due to a lack of description of the differences between imaging scanners, vendor dependencies, imaging at multiple time points with collection of individual images at other time points, reducing overfitting by reducing functionality or multiple testing, prospective registration in trial databases, comparison with the “gold standard,” and open science and data—open code and data. The validation set was generated by random sampling. Overall, the average score for the 33 studies was 7 (range: 5–10) (**Table 1**).

**Table 1:** Quality evaluation results of inclusion in the original study using RQS.

No.	Author	Year	v1	v2	v3	v4	v5	v6	v7	v8	v9	v10	v11	v12	v13	v14	v15	v16	overall
1	Elisabetta De Bernardi (17)	2018	1	0	0	0	0	1	0	0	1	0	0	2	0	0	0	0	5
2	Sigmund Ytre-Hauge (18)	2018	1	0	0	0	0	1	0	0	0	1	0	2	0	1	1	0	7
3	M. Bereby-Kahane (19)	2020	1	1	0	0	0	1	0	0	0	1	0	2	0	0	0	0	6
4	Cinzia Crivellaro (21)	2020	0	1	0	0	0	1	0	0	0	1	0	2	0	1	0	0	6
5	Yuqing Han (22)	2021	1	1	0	0	0	1	0	0	0	1	0	2	0	0	0	0	6
6	Yan Luo (23)	2020	1	1	0	0	0	1	0	1	0	1	0	2	0	0	0	0	7
7	Bi Cong Yan (24)	2020	1	1	0	0	0	1	0	1	0	1	0	5	0	1	1	0	12
8	Jingya Chen (20)	2021	1	1	0	0	0	1	0	0	0	1	0	2	0	0	0	0	6
9	Ling Long (25)	2021	1	1	0	0	0	1	0	0	0	0	0	2	0	0	0	0	5
10	Alejandro Rodríguez-Ortega (26)	2021	1	1	0	1	0	1	0	1	0	0	0	2	0	0	0	0	7
11	Çiğdem SOYDAL	2021	1	1	0	1	0	1	0	1	0	0	0	2	0	0	0	0	7
12	Arnaldo Stanzione	2020	1	1	0	1	0	1	0	0	1	0	0	2	0	0	0	0	7
13	Yuquan Xu (29)	2021	1	0	0	0	0	1	0	0	0	1	0	2	0	1	0	0	6
14	Bi Cong Yan (24)	2020	1	1	0	0	0	1	0	0	0	0	0	5	0	0	0	0	8
15	Bi Cong Yan	2021	1	1	0	0	0	1	0	1	0	1	0	2	0	0	0	0	7
16	Lan-Yan Yang (32)	2021	1	0	0	0	0	1	0	1	0	0	0	2	0	0	0	0	5
17	Kaiyue Zhang (33)	2021	0	1	0	0	0	1	0	0	1	1	0	2	0	1	1	0	8
18	Tao Zheng (34)	2021	1	1	0	0	0	1	0	0	1	0	0	2	0	0	0	0	6
19	Xiaojun Chen	2021	1	1	0	0	0	1	0	0	1	0	0	3	0	1	0	0	8
20	Thierry L. Lefebvre (36)	2022	1	1	0	0	0	1	0	0	0	0	0	2	0	0	0	0	5
21	Xue-Fei Liu (37)	2022	1	1	0	0	0	1	0	0	0	1	0	2	0	0	0	0	6
22	Pier Paolo Mainenti (38)	2022	1	0	0	0	0	1	0	0	0	0	0	3	0	0	0	0	5
23	Satoshi Otani (39)	2022	1	1	0	0	0	1	0	0	0	0	0	2	0	0	0	0	5
24	Yaoxin Wang	2022	0	1	0	0	0	1	0	0	1	1	0	2	0	1	1	0	8
25	Mingli Zhao (41)	2022	1	1	0	0	0	0	0	0	0	1	0	3	0	0	0	0	6
26	Xue-Fei Liu (42)	2022	1	1	0	0	0	1	0	1	1	1	0	2	0	1	0	0	9
27	Juan Bo (43)	2022	1	1	0	0	0	1	0	1	1	0	0	5	0	1	1	0	12
28	Qiu Bi (44)	2022	1	0	0	0	0	1	0	1	1	1	0	0	0	0	0	0	5
29	Thierry L. Lefebvre (36)	2022	1	1	0	0	0	1	0	0	1	1	0	3	0	0	0	0	8
30	Veronica Celli (46)	2022	1	1	0	0	0	1	0	0	1	0	0	3	0	1	1	0	9
31	Jieying Zhang (47)	2022	1	1	0	0	0	1	0	1	1	0	0	0	0	0	0	0	5
32	Maura Miccò (48)	2022	1	1	0	0	0	1	0	0	1	0	0	3	0	1	0	0	8
33	X.-F. Liu (49)	2023	1	1	0	0	0	1	0	1	1	0	0	0	0	1	1	0	7

### 3.4 Meta-analysis

#### 3.4.1 Preoperative diagnosis of malignant lesions

ML models based on radiomics for the diagnosis of malignant lesions predominantly employed logistic regression. In the training dataset, the pooled c-index, sensitivity, and specificity of ML models based solely on clinical features were 0.733 (95%CI: 0.674–0.791), 0.55–0.73, and 0.67–0.77, respectively. The pooled c-index, sensitivity, and specificity of ML models constructed solely using radiomics features were 0.869 (95%CI: 0.809–0.928), 0.88 (95%CI: 0.81–0.93), and 0.73 (95%CI: 0.63–0.82), respectively. The pooled c-index, sensitivity, and specificity of ML models constructed based on radiomics and clinical features were 0.924 (95%CI: 0.910–0.937), 0.83 (95%CI: 0.80–0.87), and 0.88 (95%CI: 0.85–0.90), respectively (**Tables 2**, **3**).

**Table 2:** Meta-analysis results of the c-index for radiomics-based models in the detection of malignant endometrial lesions and preoperative identification of risks in EC patients.

Modeling variable:	Detected event:	Training set:		Validation set:	
		n	c-index(95%CI)	n	c-index(95%CI)
Clinical features					
	Malignant lesions	2	0.733(0.674–0.791)	4	0.664(0.599–0.729)
	High-grade	5	0.785(0.734–0.836)	6	0.754(0.683–0.826)
	Lymph node metastasis	2	0.658(0.591–0.726)	10	0.733(0.678–0.788)
	Lymphovascular space invasion	2	0.728(0.682–0.774)	2	0.696(0.613–0.778)
	Deep myometrial invasion	3	0.727(0.669–0.786)	2	0.684(0.585–0.782)
Radiomics features					
	Malignant lesions	4	0.869(0.809–0.928)	5	0.860(0.801–0.919)
	High-grade	9	0.804(0.737–0.871)	17	0.781(0.744–0.818)
	Lymph node metastasis	5	0.799(0.733–0.864)	5	0.823(0.756–0.891)
	Lymphovascular space invasion	9	0.798(0.735–0.862)	9	0.786(0.746–0.825)
	Deep myometrial invasion	14	0.845(0.807–0.883)	11	0.818(0.781–0.854)
Radiomics + clinical features					
	Malignant lesions	4	0.924(0.910–0.937)	9	0.900(0.871–0.929)
	High-grade	5	0.936(0.910–0.962)	6	0.901(0.877–0.926)
	Lymph node metastasis	2	0.912(0.878–0.946)	6	0.906(0.882–0.929)
	Lymphovascular space invasion	2	0.850(0.778–0.923)	2	0.795(0.693–0.897)
	Deep myometrial invasion	4	0.914(0.856–0.973)	2	0.819(0.705–0.933)

n indicates the number of models.

**Table 3:** Meta-analysis results of sensitivity and specificity for radiomics-based models in the detection of malignant endometrial lesions and preoperative identification of risks in EC patients.

Modeling variable:	Detected event:	Training set:			Validation set:		
		n	sen(95%CI)	spe(95%CI)	n	sen(95%CI)	spe(95%CI)
Clinical features							
	Malignant lesions	3	0.55–0.73	0.67–0.77	4	0.67(0.48–0.82)	0.65(0.53–0.75)
	High-grade	5	0.73(0.62–0.82)	0.74(0.68–0.79)	6	0.66(0.47–0.80)	0.80(0.64–0.90)
	Lymph node metastasis	2	0.65–0.98	0.34–0.66	10	0.78(0.66–0.86)	0.75(0.65–0.83)
	Lymphovascular space invasion	2	0.68–0.98	0.37–0.71	2	0.56–0.98	0.35–0.81
	Deep myometrial invasion	2	0.69–0.91	0.38–0.69	2	0.28–0.96	0.33–0.94
Radiomics features							
	Malignant lesions	4	0.88(0.81–0.93)	0.73(0.63–0.82)	5	0.78(0.62–0.89)	0.86(0.78–0.91)
	High-grade	6	0.87(0.70–0.95)	0.80(0.71–0.86)	16	0.77(0.71–0.82)	0.76(0.62–0.86)
	Lymph node metastasis	11	0.76(0.69–0.82)	0.83(0.75–0.88)	8	0.87(0.74–0.93)	0.80(0.69–0.87)
	Lymphovascular space invasion	9	0.85(0.76–0.91)	0.76(0.66–0.84)	8	0.76(0.64–0.84)	0.76(0.65–0.84)
	Deep myometrial invasion	13	0.80(0.74–0.84)	0.81(0.76–0.86)	10	0.75(0.68–0.82)	0.81(0.73–0.88)
Radiomics + clinical features							
	Malignant lesions	6	0.83(0.80–0.87)	0.88(0.85–0.90)	9	0.82(0.77–0.86)	0.87(0.85–0.90)
	High-grade	5	0.86(0.81–0.91)	0.86(0.81–0.90)	6	0.88(0.82–0.91)	0.80(0.74–0.85)
	Lymph node metastasis	4	0.95(0.87–0.98)	0.73(0.64–0.81)	7	0.87(0.79–0.92)	0.80(0.75–0.85)
	Lymphovascular space invasion	2	0.76–0.92	0.74–0.76	2	0.56–0.63	0.75–0.96
	Deep myometrial invasion	4	0.82(0.74–0.88)	0.93(0.87–0.96)	2	0.52–0.78	0.79–0.92

In the validation dataset, the pooled c-index, sensitivity, and specificity for ML models based solely on clinical features were 0.664 (95%CI: 0.599–0.729), 0.67 (95%CI: 0.48–0.82) and 0.65 (95%CI: 0.53–0.75), respectively. The pooled c-index, sensitivity, and specificity of ML models constructed solely using radiomics features were 0.860 (95%CI: 0.801–0.919), 0.78 (95%CI: 0.62–0.89) and 0.86 (95%CI: 0.78–0.91), respectively. The pooled c-index, sensitivity, and specificity of ML models constructed based on radiomics and clinical features were 0.900 (95%CI: 0.871–0.929), 0.82 (95%CI: 0.77–0.86), and 0.87 (95%CI: 0.85–0.90), respectively (**Tables 2**, **3**).

#### 3.4.2 Diagnosis of high-grade tumors

ML models based on radiomics for the diagnosis of high-grade ECs predominantly employed logistic regression. In the training dataset, the pooled c-index, sensitivity, and specificity of ML models based solely on clinical features were 0.785 (95%CI: 0.734–0.836), 0.73 (95%CI: 0.62–0.82), and 0.74(95%CI: 0.68–0.79), respectively. The pooled c-index, sensitivity, and specificity of ML models constructed solely using radiomics features were 0.804 (95%CI: 0.737–0.871), 0.87 (95%CI: 0.70–0.95), and 0.80 (95%CI: 0.71–0.86), respectively. The pooled c-index, sensitivity, and specificity of ML models constructed based on radiomics and clinical features were 0.936 (95%CI: 0.910–0.962), 0.86 (95%CI: 0.81–0.91) and 0.86 (95%CI: 0.81–0.90), respectively (**Tables 2**, **3**).

In the validation dataset, the pooled c-index, sensitivity, and specificity of ML models based solely on clinical features were 0.754 (95%CI: 0.683–0.826), 0.66 (95%CI: 0.47–0.80) and 0.80 (95%CI: 0.64–0.90), respectively. The pooled c-index, sensitivity, and specificity of ML models constructed solely using radiomics features were 0.781 (95%CI: 0.744–0.818), 0.77 (95%CI: 0.71–0.82), and 0.76 (95%CI: 0.62–0.86), respectively. The pooled c-index, sensitivity, and specificity of ML models constructed based on radiomics and clinical features were 0.901 (95%CI: 0.877–0.926), 0.88 (95%CI: 0.82–0.91) and 0.86 (95%CI: 0.74–0.85), respectively (**Tables 2**, **3**).

#### 3.4.3 Preoperative diagnosis of lymph node metastasis

ML models utilizing radiomics for the diagnosis of lymph node metastasis predominantly employed logistic regression. In the training dataset, the pooled c-index, sensitivity, and specificity of ML models based solely on clinical features were 0.658 (95%CI: 0.591–0.726), 0.65–0.98 and 0.34–0.66, respectively. The pooled c-index, sensitivity, and specificity of ML models constructed solely using radiomics features were 0.799 (95%CI: 0.733–0.864), 0.77 (95%CI: 0.63–0.86), and 0.87 (95%CI: 0.81–0.92), respectively. The pooled c-index, sensitivity, and specificity of ML models constructed based on radiomics and clinical features were 0.912 (95%CI: 0.878–0.946), 0.92–0.95 and 0.64–0.84, respectively (**Tables 2**, **3**).

In the validation dataset, the pooled c-index, sensitivity, and specificity of ML models based solely on clinical features were 0.733 (95%CI: 0.678–0.788), 0.78 (95%CI: 0.66–0.86) and 0.75 (95%CI: 0.65–0.83), respectively. The pooled c-index, sensitivity, and specificity of ML models constructed solely using radiomics features were 0.823 (95%CI: 0.756–0.891), 0.86 (95%CI: 0.73–0.94), and 0.80 (95%CI: 0.67–0.88), respectively. The pooled c-index, sensitivity, and specificity of ML models constructed based on radiomics and clinical features were 0.906 (95%CI: 0.882–0.929), 0.87 (95%CI: 0.79–0.92) and 0.81 (95%CI: 0.75–0.85), respectively (**Tables 2**, **3**).

#### 3.4.4 Preoperative diagnosis of lymphovascular space invasion

ML models utilizing radiomics for the diagnosis of lymphovascular space invasion predominantly employed logistic regression. In the training dataset, the pooled c-index, sensitivity, and specificity of ML models based solely on clinical features were 0.728 (95%CI: 0.682–0.774), 0.68–0.98 and 0.37–0.71, respectively. The pooled c-index, sensitivity, and specificity of ML models constructed solely using radiomics features were 0.798 (95%CI: 0.735–0.862), 0.85 (95%CI: 0.76–0.91), and 0.76 (95%CI: 0.66–0.84), respectively. The pooled c-index, sensitivity, and specificity of ML models constructed based on radiomics and clinical features were 0.850 (95%CI: 0.778–0.923), 0.76–0.92 and 0.74–0.76, respectively (**Tables 2**, **3**).

In the validation dataset, the pooled c-index, sensitivity, and specificity of ML models based solely on clinical features were 0.696 (95%CI: 0.613–0.778), 0.56–0.98 and 0.35–0.81, respectively. The pooled c-index, sensitivity, and specificity of ML models constructed solely using radiomics features were 0.786 (95%CI: 0.746–0.825), 0.76 (95%CI: 0.64–0.84) and 0.76 (95%CI: 0.65–0.84), respectively. The pooled c-index, sensitivity, and specificity of ML models constructed based on radiomics and clinical features were 0.795 (95%CI: 0.693–0.897), 0.56–0.63 and 0.75–0.96, respectively (**Tables 2**, **3**).

#### 3.4.5 Preoperative diagnosis of deep myometrial invasion

ML models utilizing radiomics for the diagnosis of deep myometrial invasion predominantly employed logistic regression. In the training dataset, the pooled c-index, sensitivity, and specificity of ML models based solely on clinical features were 0.727 (95%CI: 0.669–0.786), 0.69–0.91 and 0.38–0.69, respectively. For ML models constructed solely using radiomics features, the pooled c-index, sensitivity, and specificity were 0.845 (95%CI: 0.807–0.883), 0.80 (95%CI: 0.74–0.84), and 0.81 (95%CI: 0.76–0.86), respectively. For ML models constructed based on radiomics and clinical features, the pooled c-index, sensitivity, and specificity were 0.914 (95%CI: 0.856–0.973), 0.82 (95%CI: 0.74–0.88) and 0.93 (95%CI: 0.87–0.96), respectively (**Tables 2**, **3**).

In the validation dataset, the pooled c-index, sensitivity, and specificity of ML models based solely on clinical features were 0.684 (95%CI: 0.585–0.782), 0.28–0.96 and 0.33–0.94, respectively. For ML models constructed solely using radiomics features, the pooled c-index, sensitivity, and specificity were 0.818 (95%CI: 0.781–0.854), 0.75 (95%CI: 0.68–0.82), and 0.81 (95%CI: 0.73–0.88), respectively. For ML models constructed based on radiomics and clinical features, the pooled c-index, sensitivity, and specificity were 0.819 (95%CI: 0.705–0.933), 0.52–0.78 and 0.79–0.92, respectively (**Tables 2**, **3**).

## 4 Discussion

### 4.1 Summary of the main findings

This work examined the application value of radiomics-based methods in the preoperative detection of malignant endometrial lesions, high-grade tumors, lymph node metastasis, lymphovascular space invasion, and deep myometrial invasion in EC patients. Additionally, we meta-analyzed the c-index values of ML models constructed using clinical features alone, radiomics features alone, and a combination of radiomics and clinical features, and the sensitivity and specificity at the optimal cut-off values were also meta-analyzed. Our findings demonstrate that radiomics features have shown promising accuracy in the diagnosis of malignant endometrial lesions, high-grade ECs, lymph node metastasis in EC patients, lymphovascular space invasion, and deep myometrial invasion. In particular, radiomic features combined with clinical features show a more favorable performance, yielding the best results. Importantly, no overfitting phenomenon was observed in our analysis.

### 4.2 Comparison with previous studies (other reviews)

Current non-invasive preoperative diagnosis of endometrial malignancies mainly involves ultrasound, which appears to rely on different cut-off values for endometrial thickness. A systematic review by Breijer et al. (50) focusing on the detection of endometrial malignancies in asymptomatic postmenopausal women reported a sensitivity of 0.83 (95%CI: 0.19–1.00) when using a threshold of 5 mm for endometrial thickness. However, with a threshold of 6mm, the sensitivity dropped to only 0.33 (95%CI: 0.04–0.85). Similarly, Vitale et al. (51), in their systematic review on the detection of endometrial malignancies in asymptomatic postmenopausal women, did not recommend a specific cut-off value for endometrial thickness. Instead, they suggested a range of 3.0–5.9 mm, which seemed to have higher sensitivity and specificity. Furthermore, Long et al. (52), in their systematic review focusing on endometrial malignancy detection in postmenopausal women with bleeding, had excellent sensitivity of 0.96 (95%CI: 0.92–0.98) but sacrificed specificity (0.52 (95%CI: 0.42–0.61)). These findings indicate that endometrial thickness remains an important diagnostic criterion in the ultrasound-based diagnosis of endometrial malignancies. Moreover, we also observed that certain clinical features hold significant diagnostic value in detecting EC. For example, Li et al. (53) reported in their systematic review that human epididymis protein 4 demonstrated a sensitivity of 0.71 (95%CI: 0.56–0.82) and specificity of 0.87 (95%CI: 0.80–0.92). Our systematic review demonstrated that ML models solely based on radiomics for detecting endometrial malignancies achieved a sensitivity of 0.78 (95%CI: 0.62–0.89) and specificity of 0.86 (95%CI: 0.78–0.91) in the validation set. For models constructed using radiomics and clinical features, there was a modest improvement in sensitivity (0.82, 95%CI: 0.77–0.86) and specificity (0.87, 95%CI: 0.85–0.90) in the validation set.

For preoperative risk assessment of EC, three-dimensional vaginal ultrasound, MRI, and 18F-FDG PET/CT are the main imaging modalities commonly used. They primarily contribute to the preoperative diagnosis of lymph node metastasis (54, 55), lymphovascular space invasion (56), deep myometrial invasion (56), and cervical stromal invasion (57, 58). In our study, the majority of included imaging data were derived from MRI, with only two studies utilizing 18F-FDG PET/CT for identifying lymph node metastasis. A recent systematic review by Di Donato et al. (59) on MRI in EC focused on the diagnosis of high-grade tumors, deep myometrial invasion, lymph node metastasis, and lymphovascular space invasion. There are notable differences between our findings and those reported by Di Donato et al., mainly due to a broader scope of systematic search and comprehensive consideration of the importance of clinical variables in radiomics research.

Clinical features play a significant role in existing radiomics models. Reijnen et al. (60) highlighted in their systematic review that CA125 and other clinical variables can assist in identifying lymph node metastasis in EC patients. In our study, the accuracy of models solely based on clinical features was limited in the diagnosis and risk assessment of endometrial malignancies. However, models constructed based on radiomic and clinical features showed improved diagnostic performance compared to those based solely on radiomics. This finding underscores that effective modeling variables still serve as a key factor in enhancing the accuracy of ML models. In future research, exploring efficient predictive factors remains an important direction for advancing automation diagnosis of diseases.

Additionally, in our study, the models were primarily predictive nomograms based on logistic regression, with only a limited number of ANN, SVM, and DT models. The nomograms and decision trees are highly interpretable in clinical practice. The interpretability of models is significant in clinical practice (61), particularly those constructed based on clinical features. This is because in some opaque ML models such as SVM, random forest (RF), ANNs, and deep learning (DL), it becomes challenging to assess the impact of different levels of a variable on outcome events. This poses significant challenges in developing simplified risk scoring tools. The application of deep learning models based on imaging data for automatic disease diagnosis remains a daunting challenge in radiomics research, and its scope is still limited. In this context, in addition to ensuring accuracy, better interpretability seems to be an important assessment factor in model selection in clinical practice.

### 4.3 Advantages and limitations of the study

Our study explored the value of radiomics methods for the detection of endometrial malignancies and preoperative risk assessment from a systematic review perspective for the first time. However, our study also has the following limitations. (1) Despite a systematic search, the included studies were limited in quantity for different risk outcome events, which may have somewhat restricted the interpretation of our results; (2) The quality assessment of the included studies revealed concerns about the overall quality. However, we found that RQS is a stringent radiomics evaluation tool, with some items being challenging to meet in the primary studies and not applicable to certain ML models such as RFs, ANNs, SVMs, and DL (62). This resulted in relatively lower RQS scores in previously published radiomics-related systematic reviews (63, 64); (3) In the primary studies, validation methods for the models mainly were random sampling or k-fold cross-validation, with rare external validation; (4) In the primary studies, effective measures to mitigate the risk of overfitting were rarely employed when using radiomics.

## 5 Conclusions

Radiomics-based models appear to have promising diagnostic performance in the identification of endometrial malignancies and preoperative risk assessment, but the value of clinical features should not be overlooked. However, we also observed significant biases and concerns regarding the implementation of radiomics, particularly in terms of mitigating the risk of overfitting during the research process.

## Data availability statement

The original contributions presented in the study are included in the article/**Supplementary Material**. Further inquiries can be directed to the corresponding author.

## Author contributions

JH: Conceptualization, Formal analysis, Investigation, Methodology, Writing – original draft, Writing – review & editing. YL: Conceptualization, Formal analysis, Investigation, Writing – original draft, Writing – review & editing. JL: Conceptualization, Formal analysis, Investigation, Writing – review & editing. SL: Conceptualization, Methodology, Supervision, Writing – review & editing.
