# Supplementary material for: Accuracy of radiomics in the diagnosis and preoperative high-risk assessment of endometrial cancer: a systematic review and meta-analysis
Source: Front Oncol. 2024 Jan 25;14:1334546. doi: 10.3389/fonc.2024.1334546 (PMC10853997; doi:10.3389/fonc.2024.1334546)
Supplement: Supplementary file 1 [file Table_1.docx]

# Table S1 Literature search strategy

**1.Pubmed**

| Search number | Query |
| --- | --- |
| #1 | "Endometrial Neoplasms"[Mesh] |
| #2 | (((((Endometrial Neoplasms[Title/Abstract]) OR (Endometrial Neoplasm[Title/Abstract])) OR (Neoplasm, Endometrial[Title/Abstract])) OR (Neoplasms, Endometrial[Title/Abstract])) OR (Endometrial Carcinoma[Title/Abstract])) OR (endometrial tumor[Title/Abstract]) |
| #3 | ("Endometrial Neoplasms"[Mesh]) OR ((((((Endometrial Neoplasms[Title/Abstract]) OR (Endometrial Neoplasm[Title/Abstract])) OR (Neoplasm, Endometrial[Title/Abstract])) OR (Neoplasms, Endometrial[Title/Abstract])) OR (Endometrial Carcinoma[Title/Abstract])) OR (endometrial tumor[Title/Abstract])) |
| #4 | ((((Radiomics[Title/Abstract]) OR (radiomic[Title/Abstract])) OR (radiogenomic[Title/Abstract])) OR (radiomics-based[Title/Abstract])) OR (radiomic signature[Title/Abstract]) |
| #5 | (("Endometrial Neoplasms"[Mesh]) OR ((((((Endometrial Neoplasms[Title/Abstract]) OR (Endometrial Neoplasm[Title/Abstract])) OR (Neoplasm, Endometrial[Title/Abstract])) OR (Neoplasms, Endometrial[Title/Abstract])) OR (Endometrial Carcinoma[Title/Abstract])) OR (endometrial tumor[Title/Abstract]))) AND (((((Radiomics[Title/Abstract]) OR (radiomic[Title/Abstract])) OR (radiogenomic[Title/Abstract])) OR (radiomics-based[Title/Abstract])) OR (radiomic signature[Title/Abstract])) |

**2.Cochrane**

| Search number | Query |
| --- | --- |
| #1 | MeSH descriptor: [Endometrial Neoplasms] explode all trees |
| #2 | (Endometrial Neoplasms):ti,ab,kw OR (Endometrial Neoplasm):ti,ab,kw OR (Neoplasm, Endometrial):ti,ab,kw OR (Neoplasms, Endometrial):ti,ab,kw OR (Endometrial Carcinoma):ti,ab,kw |
| #3 | (endometrium tumor):ti,ab,kw OR (endometrial tumor):ti,ab,kw OR (endometrial tumour):ti,ab,kw |
| #4 | #1 or #2 or #3 |
| #5 | (Radiomics):ti,ab,kw OR (radiomic):ti,ab,kw OR (radiogenomic):ti,ab,kw OR (radiomics-based):ti,ab,kw OR (radiomic signature):ti,ab,kw |
| #6 | #4 and #5 |

**3.Embase**

| Search number | Query |
| --- | --- |
| #1 | 'endometrium tumor'/exp |
| #2 | 'endometrium tumor':ab,ti OR 'endometrial neoplasms':ab,ti OR 'endometrial neoplasm':ab,ti OR 'neoplasms, endometrial':ab,ti OR 'endometrial carcinoma':ab,ti OR 'carcinoma, endometrial':ab,ti |
| #3 | #1 OR #2 |
| #4 | 'radiomics'/exp |
| #5 | radiomics:ab,ti OR radiomic:ab,ti OR radiogenomic:ab,ti OR 'radiomics based':ab,ti OR 'radiomic signature':ab,ti |
| #6 | #4 OR #5 |
| #7 | #3 AND #6 |

**4.Web of science**

| Search number | Query |
| --- | --- |
| #1 | Endometrial Neoplasms (Topic) or Endometrial Neoplasm (Topic) or Neoplasm, Endometrial (Topic) or Neoplasms, Endometrial (Topic) or Endometrial Carcinoma (Topic) or endometrial tumor (Topic) |
| #2 | Radiomics (Topic) or radiomic (Topic) or radiogenomic (Topic) or radiomics-based (Topic) or radiomic signature (Topic) |
| #3 | #1 AND #2 |
