# Supplementary material for: Accuracy of radiomics in the diagnosis and preoperative high-risk assessment of endometrial cancer: a systematic review and meta-analysis
Source: Front Oncol. 2024 Jan 25;14:1334546. doi: 10.3389/fonc.2024.1334546 (PMC10853997; doi:10.3389/fonc.2024.1334546)
Supplement: Supplementary file 2 [file Table_2.docx]

# Table S2 Basic characteristics of included studies

| No. | First author | Publication year | Country | Study type | Patient source | Patient age | Radiomics source | Total sample size | Number of samples in training set | Generation of validation set | Number of samples in validation set | Variable screening method | Model type | Modeling variables | Whether to construct a radiomics score |
| --- | --- | --- | --- | --- | --- | --- | --- | --- | --- | --- | --- | --- | --- | --- | --- |
| 1 | Elisabetta De Bernardi | 2018 | Italy | case-control | 1 | Taining: 66(27–86) Validation: 63(30–80) | 18F-FDG PET/CT | 115 | 86 | Random sampling | 29 | Univariate and multivariate | Neural network multivariate model | Radiomic features, clinical features | No |
| 2 | Sigmund Ytre-Hauge | 2018 | Norway | prospective cohort study | 1 | 67 (41–93) | MRI | 180 |  |  |  | Univariate and multivariate | Logistic regression | Radiomic features, clinical features+radiomic features | No |
| 3 | M. Bereby-Kahane | 2020 | France | case-control | 1 | 66 ± 11.5 | MRI | 73 | 73 |  |  | Univariate and multivariate | Logistic regression | clinical features+Radiomic features | No |
| 4 | Cinzia Crivellaro | 2019 | Italy | case-control | 1 | 63.3±10.9 | 18F- FDG PET/ CT | 167 | 167 |  |  | Iterative thresholding | Logistic regression | Radiomic features, clinical features+radiomic features | No |
| 5 | Yuqing Han | 2020 | China | case-control | 1 | 57 (31–77) | MRI | 163 | 163 | Cross-validation | 163 | LASSO | Logistic regression | Radiomic features, clinical features | No |
| 6 | Yan Luo | 2020 | China | case-control | 1 | Taining: 58.94 ± 8.72 Validation: 54.08± 9.44 | MRI | 144 | 101 | Random sampling | 43 | LASSO | Logistic regression | Radiomic features, clinical features | Yes |
| 7 | Bi Cong Yan | 2020 | China | case-control | 5 | 59±9 | MRI | 717 | 394 | External validation | 323 | LASSO | Logistic regression | Radiomic features, clinical features+radiomic features | Yes |
| 8 | Jingya Chen | 2021 | China | case-control | 1 | 57.8±9.7 | MRI | 102 | 70 | Random sampling | 32 | LASSO | Logistic regression | Clinical features clinical features+radiomic features | No |
| 9 | Ling Long | 2021 | China | case-control | 1 | 52.9±9.0 | MRI | 184 | 138 | Random sampling | 46 |  |  | Radiomic features, clinical features+radiomic features | No |
| 10 | Alejandro Rodríguez-Ortega | 2021 | Spain | case-control | 1 | 64.7 ±10.7 | MRI | 143 | 107 | Random sampling | 36 |  |  | Clinical features+radiomic features |  |
| 11 | Çiğdem SOYDAL | 2021 | Turkey | case-control | 1 | 62 ± 10.2 | 18F-FDG PET/CT | 157 |  |  |  |  |  |  |  |
| 12 | Arnaldo Stanzione | 2020 | Italy | case-control | 1 | 62.1 ± 10.8 | MRI | 54 |  | Cross-validation |  | Shapiro-Wilk |  | Radiomic features, clinical features | No |
| 13 | Yuquan Xu | 2021 | China | case-control | 1 | 53.82±3.76 | MRI | 143 | 95 | Random sampling | 48 |  | Logistic regression | Radiomic features, clinical features | No |
| 14 | Bi Cong Yan | 2020 | China | case-control | 5 | 56.6±8.8 | MRI | 622 | 622 | Cross-validation | 622 | Random forest |  | Clinical features clinical features+radiomic features | No |
| 15 | Bi Cong Yan | 2021 | China | case-control | 1 | 39±4.3 | MRI | 209 | 104 | Random sampling | 105 | LASSO | Logistic regression | Clinical features+radiomic features, clinical features | Yes |
| 16 | Lan-Yan Yang | 2021 | Taiwan | prospective cohort study | 1 | 51.2±11.6 | MRI | 236 | 165 | Random sampling | 71 |  |  | Clinical features+radiomic features, radiomic features | Yes |
| 17 | Kaiyue Zhang | 2021 | China | case-control | 1 | 56.23±8.60 | MRI | 210 | 147 | Cross-validation | 63 |  | Logistic regression | Clinical features+radiomic features, clinical features | No |
| 18 | Tao Zheng | 2021 | China | case-control | 1 |  | MRI | 358 | 250 | Random sampling | 108 | LASSO |  | Radiomic features, clinical features | No |
| 19 | Xiaojun Chen | 2021 | China | case-control | 2 | 50 | MRI | 345 | 158 | Random sampling | 78；109 | LASSO |  | Radiomic features, clinical features | No |
| 20 | Thierry L. Lefebvre, MSc | 2022 | Canada | case-control | 2 | Taining: 66± 11  Validation: 67± 12 | MRI | 157 | 94 | External validation | 63 |  | Random forest model | Clinical features+radiomic features, clinical features | No |
| 21 | Xue-Fei Liu | 2022 | Canada | case-control | 1 | 55±8.9 | MRI | 707 | 353 | Random sampling | 354 | LASSO | Logistic regression | Radiomic features, clinical features | No |
| 22 | Pier Paolo Mainenti | 2022 | Italy | case-control | 2 | Taining: 63 Validation: 60 | MRI | 133 | 104 | External validation | 29 | LASSO |  | Radiomic features, clinical features | No |
| 23 | Satoshi Otani | 2022 | Japan | case-control | 1 | Taining: 60±10.1  Validation: 55.6± 9.54 | MRI | 200 | 150 | Random sampling | 50 |  |  | Radiomic features, clinical features | No |
| 24 | Yaoxin Wang | 2022 | China | case-control | 1 | 53.13 ± 8.77 | MRI | 266 | 185 |  | 81 | LASSO | Logistic regression | Radiomic features, clinical features |  |
| 25 | Mingli Zhao | 2022 | China | case-control | 2 | 57.3±8.4 | MRI | 163 | 107 |  | 56 |  | Logistic regression | Radiomic features |  |
| 26 | Xue-Fei Liu | 2022 | China | case-control | 5 |  | MRI | 339 | 226 | Random allocation | 113 | LASSO | Logistic regression | Clinical features, radiomic features, clinical features+radiomic features | Yes |
| 27 | Juan Bo | 2022 | China | case-control | 1 | 36-76岁= | MRI | 136 | 95 | Random allocation | 41 | LASSO | Logistic regression | Clinical features, radiomic features, clinical features+radiomic features | Yes |
| 28 | Qiu Bi | 2022 | China | case-control | 2 | Taining: 50.36 ± 8.26 Validation1: 50.52 ± 9.80  Validation2: 54.32 ± 9.34 | MRI | 371 | 245 | Random allocation | internal validation (82)+ external validation (44) | LASSO | Logistic regression | Clinical features, radiomic features, clinical features+radiomic features | Yes |
| 29 | Thierry L. Lefebvre | 2022 | Canada | case-control | 2 | Training set: 65.4±10.2  Validation: 67.0±11.6 | MRI | 128 | 75 | Random allocation | 53 |  | Logistic regression | Radiomic features | No |
| 30 | Veronica Celli | 2022 | Italy | case-control | 2 | 66 ± 11.5 | MRI | 64 | Risk classification 43+42+43 Prediction of tumor uterine invasion (LVSI) 49 | Cross-validation | Risk classification 21+22+21 Prediction of tumor uterine invasion （LVSI）15 |  | Logistic regression | Radiomic features | No |
| 31 | Jieying Zhang | 2022 | China | case-control | 1 | Training: 46.7 ± 4.9 Validation Set : 47.1 ± 5.2 | MRI | 122 patients（78 AEH cases and 44 CEC cases） | 87 | Prospective validation | 35 | LASSO | Logistic regression | Radiomic features clinical features clinical features+radiomic features | Yes |
| 32 | Maura Miccò | 2022 | Italy | case-control | 2 | Training: 62(mean) Validation Set : 58(mean) | MRI | 96 | 73 | Random allocation | 23（18 LVSI cases） | LASSO | Logistic regression | Radiomic features | No |
| 33 | X.-F. Liu | 2023 | China | case-control | 1 | 56 ± 9 | MRI | 875 | 437 [Type I (350) Type II (87)] | Random allocation | 438【Type I (354) Type II (84)】 | LASSO, multivariate | Logistic regression | Clinical features, clinical features+radiomic features | Yes |
